# Supplementary material for: Turnover number predictions for kinetically uncharacterized enzymes using machine and deep learning
Source: Nat Commun. 2023 Jul 12;14:4139. doi: 10.1038/s41467-023-39840-4 (PMC10338564; doi:10.1038/s41467-023-39840-4)
Supplement: Supplementary file 1 — Supplementary Information [file 41467_2023_39840_MOESM1_ESM.pdf]

Turnover number predictions for kinetically uncharacterized enzymes using machine and deep learning

Supplementary Information

Supporting Figures 1-8

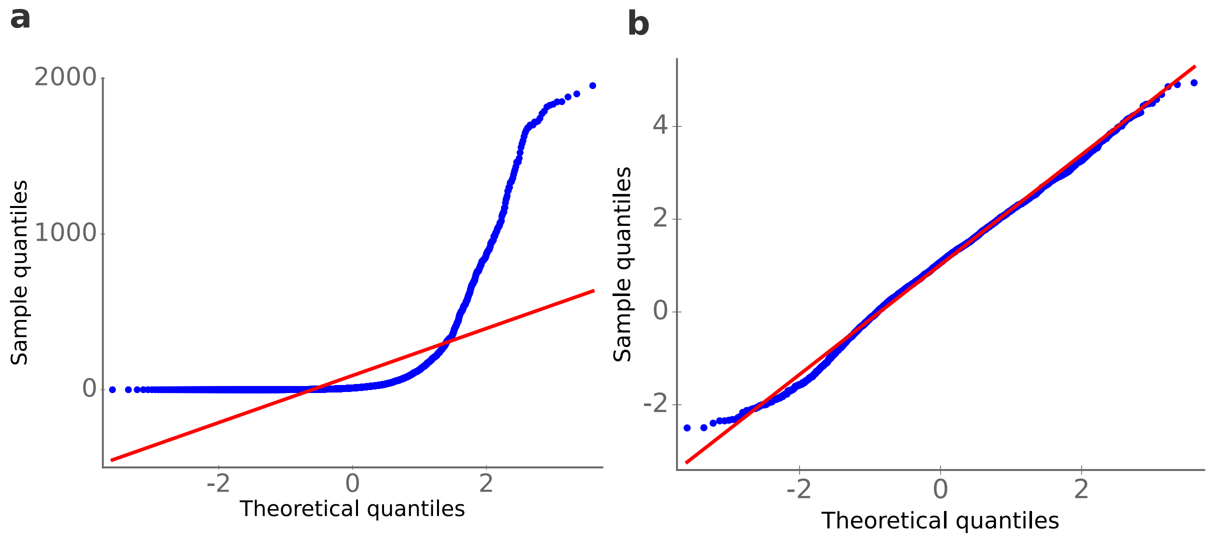

Supplementary Figure 1. Turnover numbers are approximately log-normally distributed. Q-Q plots of (**a**) untransformed values and (**b**) $\log_{10}$-transformed values. If all points fall close the red linear lines, it indicates that the data is approximately normally distributed. Source data are provided as a Source Data file.

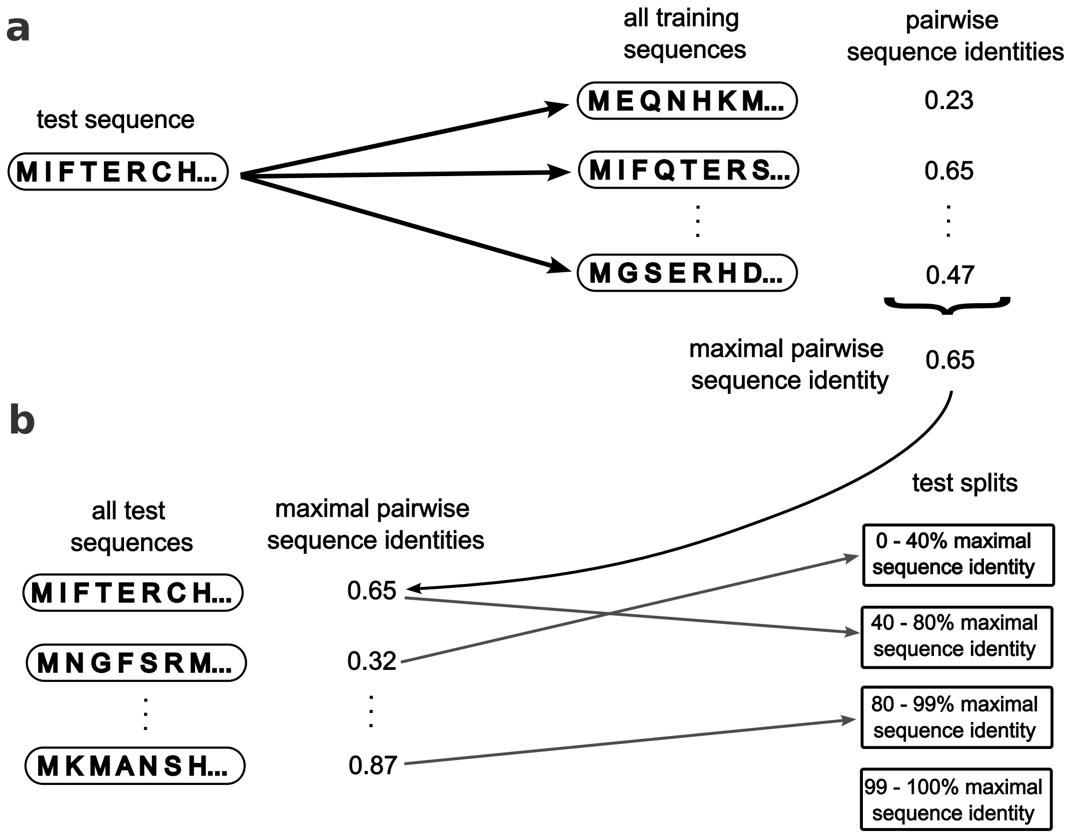

Supplementary Figure 2. Splitting the test set into different subsets according to maximal sequence identity. (a) Calculating the pairwise sequence identity for each enzyme amino acid sequence in the test set compared to all amino acid sequences in the training set. **(b)** Splitting the test set into 4 subsets according to the maximal sequence identities of the amino acid sequences.

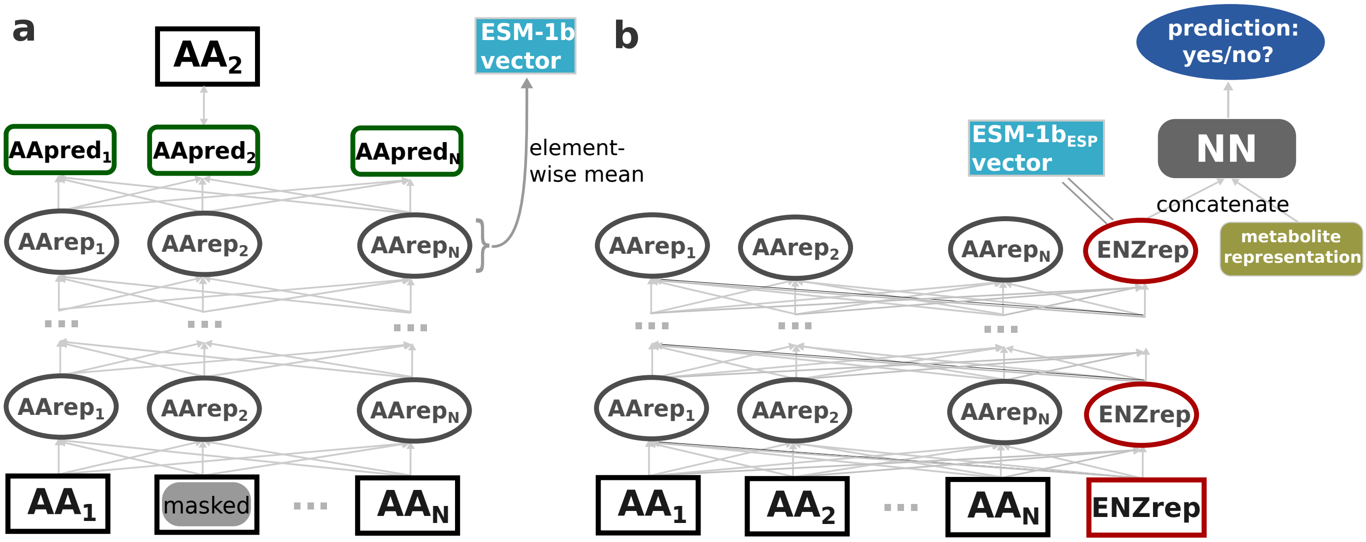

Supplementary Figure 3. Model architecture of the ESM-1b models used to calculate enzyme representations. (a) ESM-1b model. Amino acids of a protein sequence are represented with numerical vectors and passed through a transformer network. Some amino acid representations are masked. All representations are iteratively updated 33 times, using information about neighboring and distant amino acids. The ESM-1b model is trained to predict the masked amino acids. ESM-1b vectors are calculated by taking the element-wise mean of all representations in the last layer. **(b)** Modified ESM-1b model. An additional representation for the whole enzyme is added to the amino acid representations. After updating all representations 33 times, the enzyme representation is concatenated with a small molecule representation. The network is trained to predict whether the small molecule is a substrate for the given enzyme. After training, the ESM-$1b_{ESP}$ vector is extracted as the enzyme representation before adding the small molecule representation.

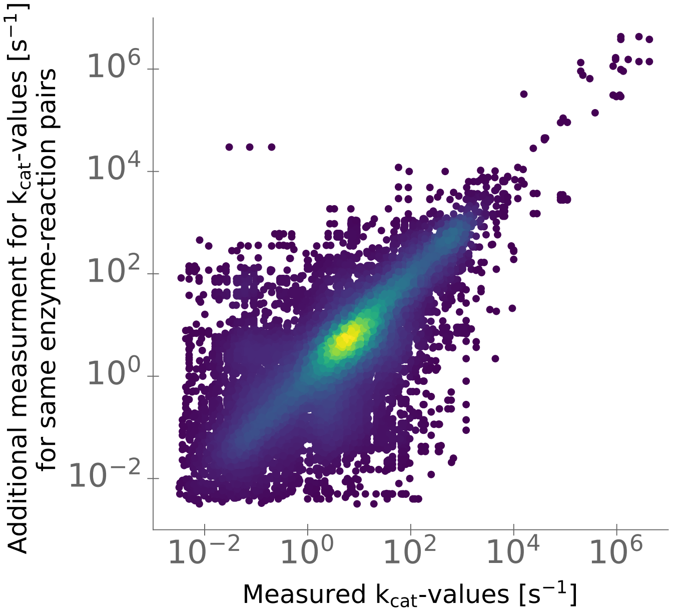

Supplementary Figure 4. Different measurements for the same enzyme-reaction pair can be very noisy. For all enzyme-reaction pairs with multiple experimentally measured values in our dataset, we plotted measured values against different measurements for the same enzyme-reaction pair. The colors indicate different densities of the data points. Lighter colors indicate a higher point density. Source data are provided as a Source Data file.

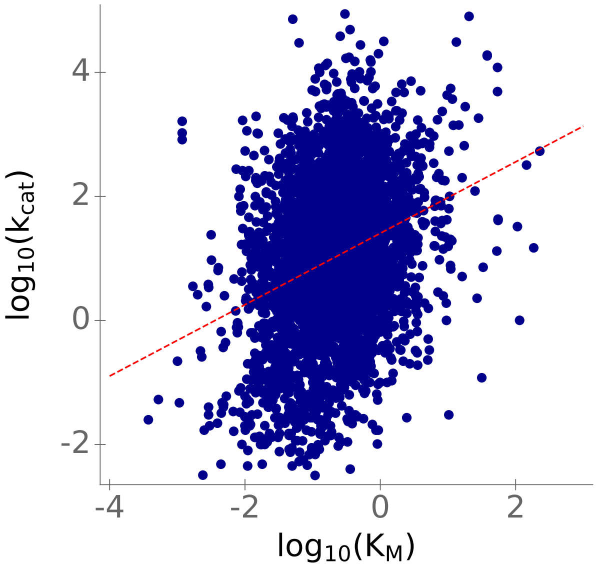

Supplementary Figure 5. Michaelis constants *KM* compared to values. We obtained either experimentally measured *KM* values from BRENDA or predicted *KM* values for every reaction and we plotted these values against the corresponding values on a $\log_{10}$-scale. The plot contains 4 271 data points from our training and test set. The red dashed line displays the correlation between both variables. Source data are provided as a Source Data file.

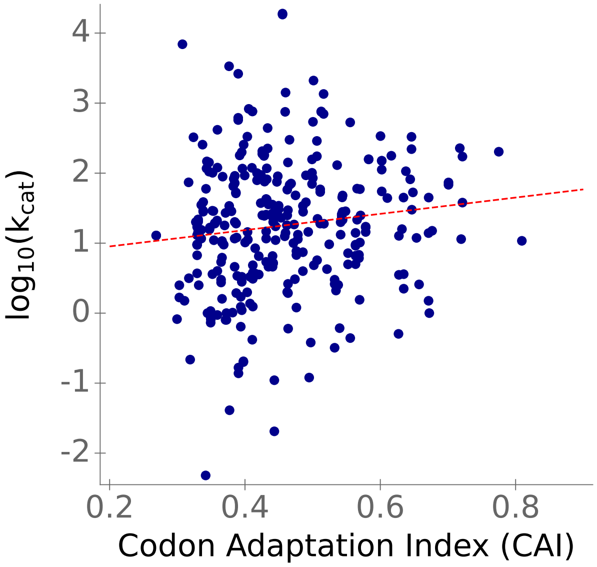

Supplementary Figure 6. Codon Adaptation Index (CAI) for enzymes from *E. coli* compared to their values. We calculated the CAI for genes from *E. coli* and plotted it against their $\log_{10}$-transformed value. The plot contains 303 data points from our training and test set. The red dashed line displays the correlation between both variables. Source data are provided as a Source Data file.

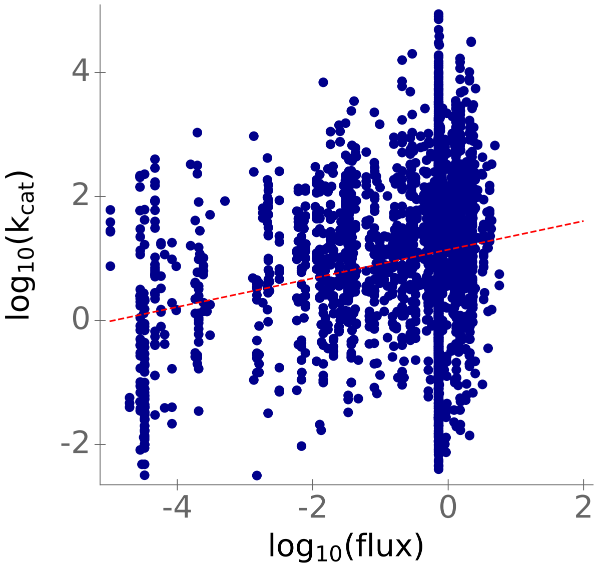

Supplementary Figure 7. Predicted reaction fluxes compared to values. We obtained either predicted reaction fluxes via parsimonious flux balance analysis (pFBA) or flux variability analysis (FVA) and we plotted these values against the corresponding values on a $\log_{10}$-scale. The plot contains 4 271 data points from our training and test set. The red dashed line displays the correlation between both variables. Source data are provided as a Source Data file.

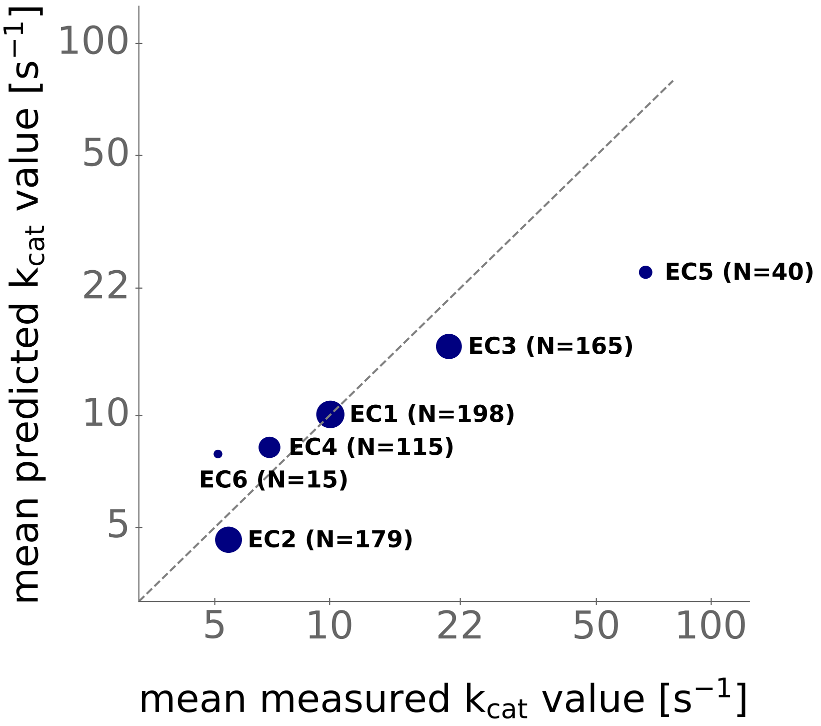

Supplementary Figure 8. Average predictions for values of different EC classes correlate with average measurements of different EC classes. We divided the test set into 6 subsets according to the first digit of the enzymes' EC numbers. We calculated the average predicted and average measured value for each of these classes. The areas of the circles are proportional to the number of predicted data points in each subset. Source data are provided as a Source Data file.

Supplementary Table 1

Supplementary Table 1. Results of four different machine learning algorithms on the test set. Hyperparameter optimizations for all models were performed with 5-fold cross-validations on the training set.

	R^2	**Pearson** r	**MSE**
**Gradient Boosting (mean)**	0.44	0.67	0.81
**Gradient Boosting (joint model input)**	0.40	0.64	0.86
**Random Forest**	0.36	0.61	0.92
**Neural Network**	0.32	0.59	0.97
**Linear Regression**	0.29	0.54	1.01
