# Supplementary material for: Turnover number predictions for kinetically uncharacterized enzymes using machine and deep learning
Source: Nat Commun. 2023 Jul 12;14:4139. doi: 10.1038/s41467-023-39840-4 (PMC10338564; doi:10.1038/s41467-023-39840-4)
Supplement: Supplementary file 3 — Reporting Summary [file 41467_2023_39840_MOESM3_ESM.pdf]

nature portfolio

Corresponding author(s): Martin Lercher

Last updated by author(s): Jun 16, 2023

Reporting Summary

Nature Portfolio wishes to improve the reproducibility of the work that we publish. This form provides structure for consistency and transparency in reporting. For further information on Nature Portfolio policies, see our Editorial Policies and the Editorial Policy Checklist.

Statistics

For all statistical analyses, confirm that the following items are present in the figure legend, table legend, main text, or Methods section.

n/a	Confirmed	
☐	☒	The exact sample size (*n*) for each experimental group/condition, given as a discrete number and unit of measurement
☒	☐	A statement on whether measurements were taken from distinct samples or whether the same sample was measured repeatedly
☐	☒	The statistical test(s) used AND whether they are one- or two-sided<br>*Only common tests should be described solely by name; describe more complex techniques in the Methods section.*
☐	☒	A description of all covariates tested
☒	☐	A description of any assumptions or corrections, such as tests of normality and adjustment for multiple comparisons
☐	☒	A full description of the statistical parameters including central tendency (e.g. means) or other basic estimates (e.g. regression coefficient) AND variation (e.g. standard deviation) or associated estimates of uncertainty (e.g. confidence intervals)
☐	☒	For null hypothesis testing, the test statistic (e.g. *F*, *t*, *r*) with confidence intervals, effect sizes, degrees of freedom and *P* value noted<br>*Give P values as exact values whenever suitable.*
☒	☐	For Bayesian analysis, information on the choice of priors and Markov chain Monte Carlo settings
☒	☐	For hierarchical and complex designs, identification of the appropriate level for tests and full reporting of outcomes
☐	☒	Estimates of effect sizes (e.g. Cohen's *d*, Pearson's *r*), indicating how they were calculated

Our web collection on statistics for biologists contains articles on many of the points above.

Software and code

Policy information about availability of computer code

Data collection	We used Python 3.7 to collect the data. All code and all used Python packages are available on our GitHub repository https://github.com/AlexanderKroll/kcat_prediction.
Data analysis	All software was coded in Python 3.7. We used the software package EMBOSS 6.6.0 to calculate pairwise sequence identities between protein amino acid sequences. We created enzyme representations using the deep learning library PyTorch, and we fitted the gradient boosting models using the library XGBoost. The code used to generate the results, in the form of Jupyter notebooks, is available at https://github.com/AlexanderKroll/kcat_prediction.

For manuscripts utilizing custom algorithms or software that are central to the research but not yet described in published literature, software must be made available to editors and reviewers. We strongly encourage code deposition in a community repository (e.g. GitHub). See the Nature Portfolio guidelines for submitting code & software for further information.

Data

Policy information about availability of data

All manuscripts must include a data availability statement. This statement should provide the following information, where applicable:

- Accession codes, unique identifiers, or web links for publicly available datasets
- A description of any restrictions on data availability
- For clinical datasets or third party data, please ensure that the statement adheres to our policy

We used the BRENDA, UniProt, and Sabio-RK databases to create a kcat dataset. All datasets that we downloaded and created to generate the results of the

manuscript are available at https://doi.org/10.5281/zenodo.7849347.

Research involving human participants, their data, or biological material

Policy information about studies with human participants or human data. See also policy information about sex, gender (identity/presentation), and sexual orientation and race, ethnicity and racism.

Reporting on sex and gender	Not applicable
Reporting on race, ethnicity, or other socially relevant groupings	Not applicable
Population characteristics	Not applicable
Recruitment	Not applicable
Ethics oversight	Not applicable

Note that full information on the approval of the study protocol must also be provided in the manuscript.

Field-specific reporting

Please select the one below that is the best fit for your research. If you are not sure, read the appropriate sections before making your selection.

☒ Life sciences ☐ Behavioural & social sciences ☐ Ecological, evolutionary & environmental sciences

For a reference copy of the document with all sections, see nature.com/documents/nr-reporting-summary-flat.pdf

Life sciences study design

All studies must disclose on these points even when the disclosure is negative.

Sample size	Sample size was determined by the availability in the databases used.
Data exclusions	We hypothesized that we do not have enough data to train a model to predict the catalytic effect of enzyme mutations or to predict the kcat value of non-natural enzyme-reaction pairs, which have not been shaped by natural selection. Hence, we removed all data points with non-wild-type enzymes and all non-natural reactions (see Methods, "Data preprocessing")
Replication	The training and validation of our machine learning models can be reproduced using the code available at our GitHub repository.
Randomization	We split the dataset into 80% training data and 20% test data in such a way that enzymes with the same amino acid sequence would not occur both in the training and in the test set. We further split the training set into 5 disjoint subsets to perform 5-fold cross validations (CVs) for hyperparameter optimization of our machine learning models. The CV sets were also constructed in such a way that no two subsets contained enzymes with identical amino acid sequences.
Blinding	Blinding was not necessary for the training and validation of machine learning models.

Reporting for specific materials, systems and methods

We require information from authors about some types of materials, experimental systems and methods used in many studies. Here, indicate whether each material, system or method listed is relevant to your study. If you are not sure if a list item applies to your research, read the appropriate section before selecting a response.

Materials & experimental systems

n/a	Involved in the study	
☒	☐	Antibodies
☒	☐	Eukaryotic cell lines
☒	☐	Palaeontology and archaeology
☒	☐	Animals and other organisms
☒	☐	Clinical data
☒	☐	Dual use research of concern
☒	☐	Plants

Methods

n/a	Involved in the study	
☒	☐	ChIP-seq
☒	☐	Flow cytometry
☒	☐	MRI-based neuroimaging
